# Natural Polysaccharide-Based Hydrogels Used for Dye Removal

**DOI:** 10.3390/gels10040243

**Published:** 2024-04-02

**Authors:** Magdalena-Cristina Stanciu, Carmen-Alice Teacă

**Affiliations:** 1 Natural Polymers, Bioactive and Biocompatible Materials Department, “Petru Poni” Institute of Macromolecular Chemistry, 41A, Gr. Ghica-Voda Alley, 700487 Iasi, Romania; 2 Center for Advanced Research in Bionanoconjugates and Biopolymers, “Petru Poni” Institute of Macromolecular Chemistry, 41A, Gr. Ghica-Voda Alley, 700487 Iasi, Romania

**Keywords:** hydrogels, natural polysaccharides, dye removal

## Abstract

Removal of contaminants from discharge water is vital and demands urgent assistance with the goal to keep clean water. Adsorption is one of the most common, efficient, and low-priced methods used in water treatment. Various polysaccharide-based gels have been used as efficient dye adsorbents from wastewater. This review summarizes cutting-edge research of the last decade of different hydrogels based on natural polysaccharides (chitin, chitosan, cellulose, starch, pullulan, and dextran) concerning their dye adsorption efficiency. Beyond their natural abundance, attributes of polysaccharides such as biocompatibility, biodegradability, and low cost make them not only efficient, but also environmentally sustainable candidates for water purification. The synthesis and dye removal performance together with the effect of diverse factors on gels retaining ability, kinetic, and isotherm models encountered in adsorption studies, are introduced. Thermodynamic parameters, sorbent recycling capacity along with conclusions and future prospects are also presented.

## 1. Introduction

Water constitutes the primary element of the Earth and is essential for the survival of all living organisms, including plants, animals, and humans. Various hazardous chemical compounds, originating from diverse industries such as paper, textile, and plastics, are released into water, resulting in a significant volume of contaminated wastewater. Consequently, the imperative task of eliminating dangerous contaminants from water arises to uphold the integrity of pure water resources.

Among the various types of wastewater, dyeing wastewater holds a notable proportion because of the expansion of dye manufacturing. Dyes, substances imparting color to textiles, leather, paper, plastics, and rubber, contribute significantly to this type of wastewater [1,2,3,4,5]. Presently, the Colour Index records the usage of over 10,000 different dyes and pigments in industries. The substantial presence of these dyes in wastewater can impede sunlight transmission in rivers, adversely affecting the photosynthetic activities of aquatic species and leading to a decline in O_2_ levels in water. Additionally, dyes contain poisonous products, including traces of heavy metals (Cu, Pb, Co, Cd), aromatics, and amines. Of grave concern is the fact that dyes exhibit mutagenic, teratogenic, and carcinogenic effects, causing dysfunction in various human organs. Moreover, exposure to dyes can result in dermatitis, rhinitis, skin rash, asthma, or various tissue modifications. Regrettably, these synthetic products pose significant challenges in terms of degradation, displaying a strong chemical stability under conditions of light, heat, or exposure to oxidizing agents, with many of them having a half-life extending over several years [6].

Categorized by their origin, dyes can be classified into two main types: natural and synthetic dyes.

**Figure 1:**
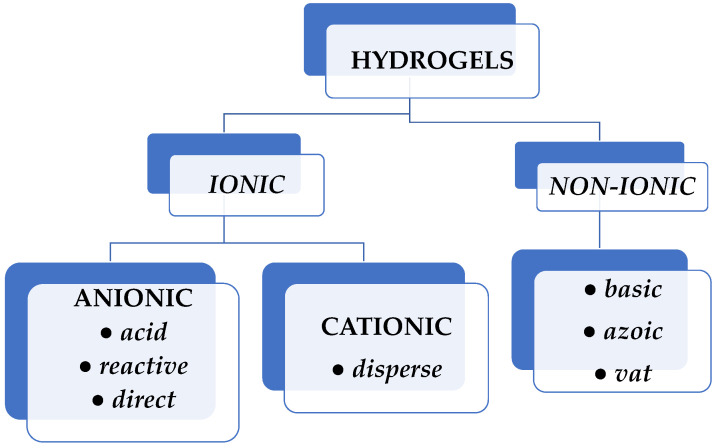
Classification of synthetic dyes according to their electrical charge.

Natural dyes are sourced from natural elements such as plants, invertebrates, or minerals. Most natural dyes are made from plant components such as roots, berries, bark, leaves, and wood, as well as from other biological microorganisms such as fungi. Synthetic dyes were developed and supplanted natural dyes, particularly in the textile industry. Based on their electrical charge, synthetic dyes can be ionic or nonionic. Anionic dyes (reactive, direct, and acid type) and cationic dyes (disperse type) exhibit good solubility in water, whereas nonionic dyes (basic, azoic, and vat dyes) do not have this feature (Figure 1) [7]. 

Due to the unfavorable results and prolonged persistence in water, it is imperative to use treatment methods and technologies for the elimination of dyes from sewage. Treating methods encompass physical separation techniques such as filtration, sedimentation, distillation, and adsorption, as well as chemical processes such as chlorination, flocculation, and coagulation, oxidation, ion-exchange, ozonation, the use of electromagnetic radiation (UV light), and biological processes, such as slow sand filters or biologically active carbon [8,9].

**Figure 2:**
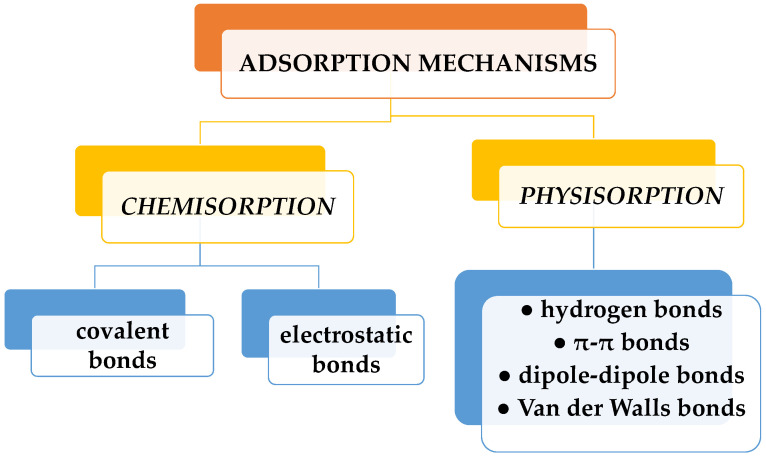
Types of dye adsorption mechanisms.

Among these methods, the adsorption process stands out as the optimal alternative. It is a straightforward and cost-effective way with a brief analysis time and the generation of harmless secondary compounds. Because of its great efficacy and the potential for regenerating the adsorbent for multiple reuses, adsorption is widely recognized as one of the most popular and utilized treatment methods for dye removal [10,11]. Dye sorption can be physical or chemical (Figure 2). Physisorption is the process whereby dye molecules are adsorbed to a sorbent surface by weak forces (hydrogen bonds, dipole-dipole bonds, van der Waals, or π-π bonds). Chemisorption is the sorption achieved by chemical bonding (covalent or electrostatic). While physisorption can be reversed by the variation of different factors (heat, pH, pressure, etc.), chemisorption cannot be. Dyes are adsorbed at an active site on the surface of the adsorbent through electrostatic interaction, ion exchange, complexation, and/or chelation. Solution pH, initial dye concentration, functional groups, and adsorbent dosage in particular are external factors that mainly affect dye adsorption processes. The profitability of the adsorption process is given by the price of the sorbent and its efficiency. Thus, activated carbon, widely employed in water purification, is an excellent adsorbent, but its main drawback lies in its high cost [12]. It is used in pretreatment processes for the elimination of free chlorine and chloramines, and also serves to eliminate trace organic impurities from purified water. Instead, other sorbents, used in elimination of dyes in water, have a low price. These include unconventional materials such as clay products (bentonite, kaolinite, smectite, montmorillonite), zeolites, siliceous materials (alunite, perlite, sepiolite, attapulgite, silica beads), agricultural wastes such as peels, leaves, and seeds (bagasse pith, wheat straw, rice husk, sawdust, bark, sugarcane bagasse, cotton fiber), industrial waste items (waste carbon slurries, fly ash, red mud), and biomass (algae, fungi, bacteria). Hydrogels are polymeric materials most often used in dye adsorption, due to their structural characteristics. 

## 2. Hydrogels

**Figure 3:**
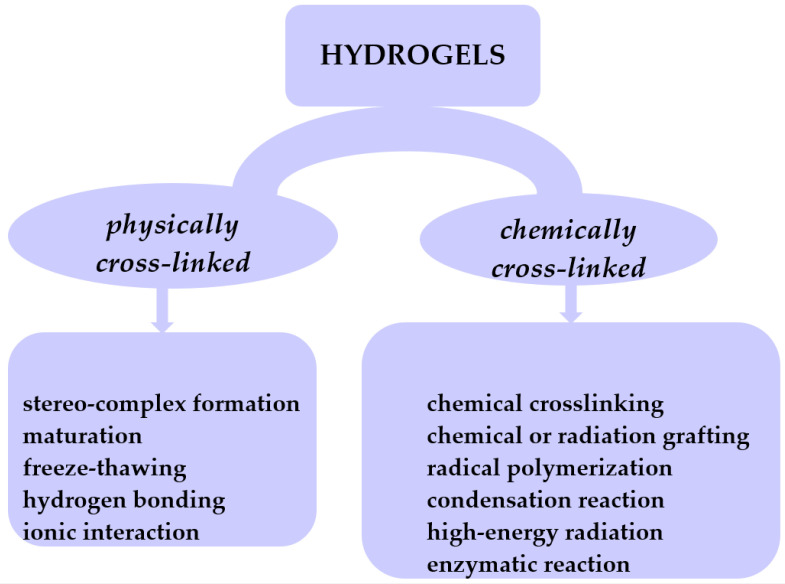
Classification of hydrogels based on their cross-linking mechanism.

Hydrogels are hydrophilic cross-linked polymers having a 3D network which remains insoluble in any solvent. These structures can absorb water or biological fluids and undergo a swelling process through their pores [13,14,15,16,17,18]. This places gels in a state that lies between that of a solid and a liquid. The cross-links impart distinctive characteristics to hydrogels, allowing them to exhibit reversible changes such as swelling and deswelling. In the dehydrated state, hydrogels undergo contraction and revert to their initial volume. Typically, these polymers preserve a substantial portion of water within their 3D network. However, there are gels known as superabsorbents that have the capacity to absorb water ranging from 1000 to 100,000%. Hydrogels can be categorized based on various criteria. Nevertheless, given that hydrogels are fundamentally formed through cross-linking networks, their classification based on the cross-linking mechanism leads to two categories: physically cross-linked and chemically cross-linked hydrogels (Figure 3). 

Physically cross-linked hydrogels can be obtained through methods such as freeze–thawing [19], stereocomplex formation [20], ionic interaction [21,22], H-bonding [23,24], and maturation (heat-induced aggregation) [25]. Chemically cross-linked hydrogels can be achieved through various methods such as chemical cross-linking [26], chemical or radiation grafting [27], radical polymerization [28], condensation reaction [29,30], enzymatic reaction [31], and high-energy radiation [32,33]. Depending on their physical state, hydrogels are classified into solids, semisolids, and liquids. Considering polymeric composition, hydrogels can be classified as homo-polymeric, co-polymeric, and interpenetrating polymeric hydrogel-IPN (semi or full). In terms of network electrical charge, hydrogels are categorized as nonionic, ionic (cationic or anionic), amphoteric electrolyte (possessing both acidic and basic groups), and zwitterion with anionic and cationic groups in every polymeric unit. Based on starting materials, hydrogels are classified into natural, synthetic, or hybrid, which combine both natural and synthetic gel components. Pure gels exhibit limited mechanical and thermal stability. The incorporation of nanofillers, such as graphene oxide (GO), carbon clay, bentonite (BNTN), hydroxyapatite (HAp), or montmorillonite (MMT), into the gel matrix results in the creation of novel products known as nanocomposites, endowed with enhanced properties.

Polymeric hydrogels have diverse applications across multiple fields, including medicine, cosmetics, agriculture, and materials science. In the medical sector, they play a crucial role in drug delivery systems [34], wound dressings [35], tissue engineering [36], or as agents with antibacterial properties [37]. Within cosmetics, polymeric hydrogels are employed into products such as gels, creams, and lotions to enhance texture and stability [38,39]. Moreover, in agriculture, these hydrogels may be utilized for the controlled delivery of fertilizers or pesticides [40].

### 2.1. Hydrogels Based on Natural Polysaccharides Used in Dye Adsorption

Polysaccharides are a significant class of biopolymeric materials which are stable, abundant, nontoxic, and biodegradable. Polysaccharide-based materials, displayed in a variety of forms such as hydrogels, membranes, beads/resins, or films, find widespread applications in wastewater treatment. The presence of numerous hydrophilic functional groups in their chemical structure enables the effective adsorption of dyes [41], biosorption being, in that case, a competitive, effective, and cheap approach. 

Chemical structure and characteristics of different natural polysaccharides, used in the obtaining of various hydrogels utilized for dye elimination, are listed in Table 1. 

Cross-linking and grafting of native polysaccharides are great alternatives for constructing adsorption systems with enhanced mechanical and thermal properties. Further, the incorporation of nanofillers (BNTN, GO, HAp, MMT, etc.) or magnetic nanoparticles in a gel’s structure enhanced dye sorption properties. 

**Figure 4:**
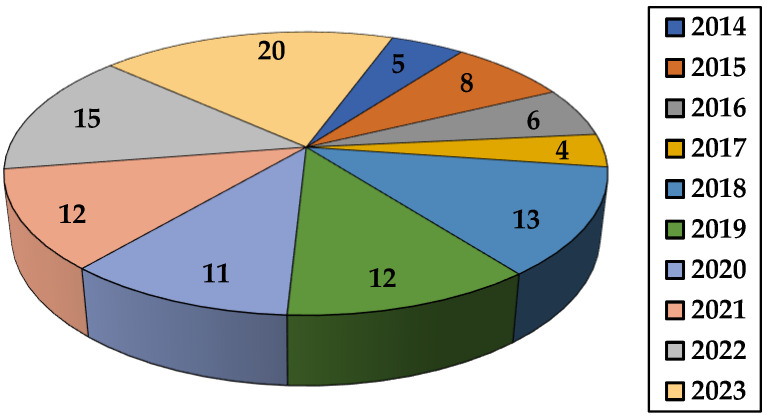
The number of papers concerning dye removal by natural polysaccharide-based hydrogels (2014–2023) (source Web of Science).

This review summarizes the studies appearing in the last decade on the application of different natural polysaccharide-based hydrogels in dye removal. Thus, synthesis and dye elimination performance of various gels based on the polysaccharides listed in Table 1 are described. Other topics covered in this paper include the impact of various factors on the gel’s ability to retain dyes, kinetic and isotherm models, as well as thermodynamic parameters met in adsorption research. This review also presents sorbent’s recycling capacity together with conclusions and suggestions for future research directions. In the last decade, the number of publications for environmentally friendly adsorption process has increased due to the increased interest shown by researchers (Figure 4).

#### 2.1.1. Chitosan and Chitin-Based Hydrogels

Chitosan (CS) (Table 1), a linear polysaccharide, is composed of D-glucosamine and N-acetyl-D-glucosamine units randomly distributed within its chemical structure, linked together by β-(1→4) bonds [48]. CS is obtained through the N-deacetylation of chitin. The inherent characteristics of CS and its derivatives, such as hydrophilicity, biocompatibility, biodegradability, and bioadhesivity, make them suitable for various applications. These include drug delivery, biomedical research, biotechnology, catalysis, the cosmetic industry, textiles, the paper industry, and enology.

Polymers based on CS are usually used in wastewater management because of their exceptional capability to adsorb dyes, cost efficiency, and adaptability in manufacturing, and also due to their mechanical and thermal stability [49] (Table 2). CS’s ability to eliminate dyes is restricted by its crystalline structure. To improve polysaccharidic selectivity and effectiveness, functional groups were attached to CS via its amino groups (C-2), as well as primary (C-6) and secondary (C-3) hydroxyl groups (Table 1). Thus, grafting and cross-linking reactions with different reagents (acrylamide, acrylic acid, N-vinylimidazole, polyvinylamine, polyacrylate triethylenetetramine, 2-acrylamido-propanesulphonic acid) presented in different papers of the last decade concerning dye sorption by hydrogels based on CS enhance the adsorption and mechanical properties, as well as chemical stability, of the native polysaccharide. The same literature showed that the addition of various fillers (GO, MMT, phytic acid) or magnetic nanoparticles (Fe_3_O_4_) in the chemical structure of above-mentioned CS-based hydrogels augmented their dye sorption properties [50,51,52,53,54,55,56,57,58,59,60,61,62,63,64]. 

A sorbent based on CS was obtained using free radical cross-link copolymerization of the polysaccharide with acrylamide (AM) and acrylic acid (AA) [50]. This process was conducted in the presence of ceric ammonium nitrate/ascorbic acid as initiator and N, N-methylene bisacrylamide (MBA) as cross-linking agent. The resulting sorbent was employed for the removal of Methyl Orange (MO). Additionally, the cross-linked copolymer exhibited notable antibacterial activity against *Pseudomonas aeruginosa*, *Escherichia coli*, and *Staphylococcus aureus*, demonstrating a high decrease in growth compared to the pristine CS. 

It is known that magnetic CS microspheres demonstrated rapid separation under a magnetic field and efficient regeneration under acidic conditions. Thus, magnetic hydrogels containing CS grafted with poly(2-acrylamido-2-methylpropane sulfonic acid) (PAMPS) was utilized for the adsorption of cationic Methylene Blue (MB) [51]. The synthesis of magnetic microspheres involved the incorporation of magnetic Fe_3_O_4_ and SiO_2_ nanoparticles into the polysaccharide matrix, followed by the grafting of PAMPS by free radical polymerization. The interaction between the magnetic grafted CS and MB dye involved both electrostatic and hydrophobic forces. Composite hydrogels based on CS have been assiduously studied in different applications. The predominant features of these composites, such as their expansive surface area and numerous functional groups, contribute favorably to an increased affinity for dye molecules, which determined their substantial adsorption efficiency. 

MMT, a soft phyllosilicate group of minerals with nanolayered structure, was often employed as a dye adsorbent due to its high swelling and cation exchange capacity. Thus, an intercalated composite of CS and MMT, acquired by transferring the polysaccharide solution to MMT suspension and heating the mixture at 70 °C, was employed to remove Reactive red 136 (RR 136) [52]. The composite’s reactive functional groups (hydroxyl, amide, amino, siloxane) played an important role in the sorption process, which was accomplished both by surface adsorption and intercalation. CS-based composite displayed excellent adsorption results even after undergoing 15 adsorption–desorption cycles.

**Figure 5:**
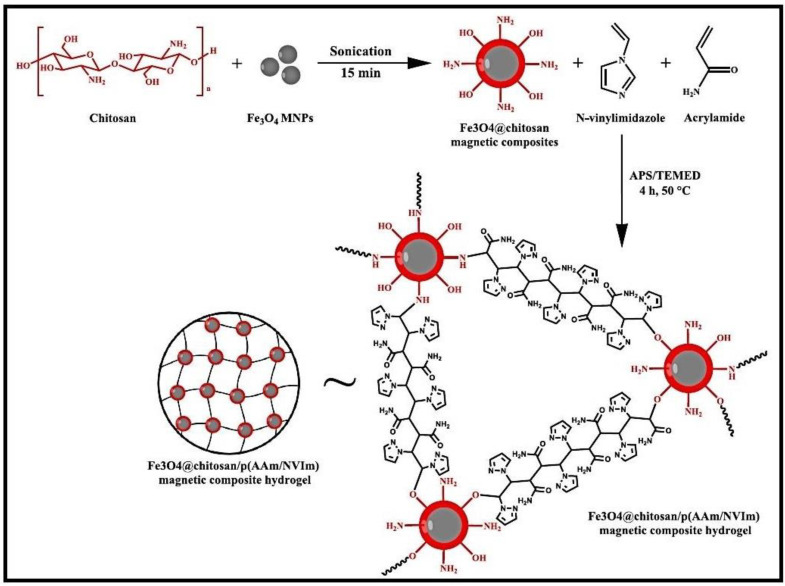
Plausible reaction scheme and structure of Fe_3_O_4_@CS/p(AAM/NVIm) magnetic composite. Reproduced with permission from [53].

A composite hydrogel, Fe_3_O_4_@CS/p(AAM/NVIm), created by the incorporation of magnetic nanoparticles Fe_3_O_4_ in CS grafted with AM and N-vinylimidazole (NVIm) was tested as adsorbent for MB dye (Figure 5) [53]. The composite hydrogel proved to be a promising recyclable, durable, robust, and efficient adsorbent for MB adsorption from wastewater.

Different amounts of phytic acid, also known as inositol hexaphosphate (IP6), were used to obtain a series of carboxymethyl CS (CMCS)/IP6 composite hydrogels, aimed to remove MO and Congo Red (CR) dyes from water [54]. CMCS/IP6 composite hydrogel, having a 3:1 molar ratio of the components, exhibited the highest adsorption capacity for MO and CR dyes at pH 7 and r.t. Furthermore, the composite demonstrated excellent reusability, stability, and swelling capacity.

A dual-network composite hydrogel with exceptional mechanical characteristics, high reusability, and significant adsorption capacity, created by incorporating CS-cross-linked polyvinyl amine (PVAm) into a cross-linked polyacrylic acid (PAA) network, was used for MB removal from aqueous solution [60]. The adsorption mechanisms were determined to be based on hydrogen bonds and electrostatic interactions between the functional groups of the hydrogel and dye molecules, from the Langmuir equation and pseudo-second-order kinetic model fitted with experimental data. The hydrogel demonstrated good reusability, with an adsorption efficiency higher than 85% for five consecutive cycles. 

A composite hydrogel having spherical structures, obtained by CS encapsulation in an organic-inorganic iron and terephthalate ligands, was used to adsorb CR dye [61]. The main mechanism of adsorption was through ionic interactions and hydrogen bonding. The adsorption followed a pseudo-first-order kinetic model and Liu isotherm model. The best performance was observed at neutral pH and r.t., the adsorption capacity being 99.97% at equilibrium. After three adsorption cycles, the recovery rate was approximately 85%. 

GO is a unique compound composed of a single layer of graphite with different oxygen-comprising capabilities [62]. GO possesses excellent properties including an increased specific surface area, strong mechanical hardiness, and good electrical and thermal conductivity. It is also resistant to corrosion. When combined with natural or synthetic hydrogels, multilayered GO sheets form composites with improved adsorption properties due to electrostatic and hydrogen bonding interactions with sorbates. The presence of epoxide and hydroxyl groups on the GO basal planes, as well as carboxyl and carbonyl groups at the edges, allows for easy diffusion of water molecules between the nanosheets’ layers, which clarifies their great dye sorbing capability. 

A hybrid sorbent consisting of CS and GO, previously treated with triethylenetetramine (TETA), was utilized for the removal of C.I. Reactive Blue 221 (RB 221) dye [63]. The occurrence of TETA-GO in the hybrid polymer equally enabled π-π bonds and ionic attraction with the reactive dyes, and also maintained a great endurance of the sorbent to acidic and alkaline media. 

A hydrogel composite made of CS, polyacrylate (PA), and GO was evaluated for its effectiveness in removing Food yellow 3 (FY 3) and MB dyes [64]. The process involved mixing PA, CS, and deionized water in a reactor to create a partially soluble slurry. GO was then introduced into the mixture, and the entire mixture was transformed into a composite hydrogel using sol-gel conversion with the help of acetic acid vapor. The addition of PA and GO significantly enhanced the swelling and mechanical properties of the composite hydrogel. Dyes were retained onto the CS-based composite by hydrogen bonding, ionic bonding, and covalent bonding. 

Chitin, the second most abundant natural biopolymer, is derived from the exoskeleton of crustaceans (such as crabs and shrimps), mollusk cartilages, and fungal cell walls (Table 1). It consists of N-acetyl-D-glucosamine units linked together with β-(1→4) bonds. Chitin possesses properties such as biocompatibility, biodegradability, affordability, and reusability. Grafting and cross-linking of pristine chitin afforded the obtaining of hydrogels with enhanced adsorption properties compared to the starting polysaccharide. Literature from the last decade showed that the introduction of different fillers (GO, r-GO tannic acid) in chitin-based gels have been utilized successfully in dye removal processes due to the enhanced adsorption properties of obtained composites [65,66] (Table 2). 

Chitin/GO hybrid gels, having different molar ratios of the components, were used as biosorbents of Neutral Red (NR) [67]. Both dyes showed an optimum adsorption at a chitin/GO molar ration of 3/1, and at an adsorption pH of 5.0 for the first dye and 4.0 for the last one, while desorption pH values were 8.0 and 9.0, respectively. 

**Figure 6:**
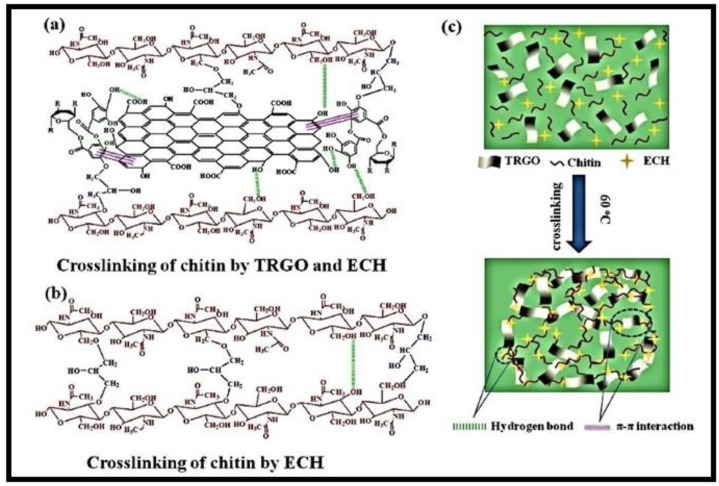
(**a**–**c**) means the syntheses of chitin-TA modified with rGO (chitin-TRGO) composite hydrogels. Reproduced with permission from [68].

A composite hydrogel (chitin-TRGO), made from chitin, tannic acid (TA), and modified with reduced graphene oxide (rGO) through a freezing–thawing process, was used to remove CR dye (Figure 6) [68]. TA served as both a reducing agent and a surface modifier for rGO. The hydroxyl groups of chitin and the phenolic hydroxyl groups of TA interacted with rGO surface through π–π interactions, resulting in a cross-linking reaction with epichlorohydrin (ECH). The resulting composite showed improved mechanical strength and enhanced sorption properties in the formation of novel materials known as nanocomposites, endowed with enhanced properties. 

**Table 2:** Dye removal performances of some CS and chitin-based hydrogels.

Adsorbent	Dye	Dye Retention/Elimination Capacity	Reference
CS-g-P(AM-co-AA)	MO	90% removal efficacy	[50]
Fe_3_O_4_, SiO_2_/CS–g-PAMPS	MB	1000 mg/L	[51]
CS-g-EDA/CS-g-MA	CR	1607 (mg/g) 1143 (mg/g)	[55]
CS-g-P(AM-co-NaMA)	Fuchsin	97.2% removal efficacy	[56]
CS/MMT	RR 136	473 mg/g	[52]
Fe_3_O_4_@CS/P(AAM/NVIm)	MB	860 mg/g	[53]
CS/Ag-HAp	RhB	127.61 mg/g	[57]
CMC/IP_6_	MO	13.62 mg/g	[54]
	CR	8.49 mg/g	
CS-gelatin/ZnO	CR	90.8% photocatalytic activity	[58]
CS/PVA/PAA	MB	596.14 mg/g	[60]
CS/iron and terephthalate ligands	CR	590.8 mg/g	[61]
CS/nano-ZnO	RB 5	189.44 mg/g	[59]
CS/PA/GO	FY 3	296.5 ± 31.7(mg/g)	[64]
	MB	280.3 ± 23.9 (mg/g)	
CS/TETA-GO	RB 221	54.2 mg/g (pH = 1) 34.8 mg/g (pH = 11)	[63]
chitin/GO	NR	57 × 10^−2^ mmol/g	[67]
chitin-TRGO	CR	230.5 mg/g	[68]

#### 2.1.2. Cellulose-Based Hydrogels

Cellulose, with the chemical formula (C_6_H_10_O_5_)_n_, is the most predominant biomaterial worldwide (Table 1). It consists of glucose units connected through β-1,4 linkages, and its polymerization degree ranges from several hundreds to tens of thousands.

Typically, plants are responsible for synthesizing cellulose, but certain bacteria can also produce it. The chains of cellulose are compressed into microfibrils, which are held together by intramolecular hydrogen bonds formed between the three hydroxyl reactive groups of each polysaccharide unit. This biocompatible and biodegradable polysaccharide has an irregular and fibred structure and is not soluble in water. Compared to other polysaccharides, cellulose has high mechanical properties. Suitable solvents for cellulose include alkali/urea (thiourea), LiCl/dimethylacetamide, and N-methyl morpholine-N-oxide. The hydroxyl reactive groups present in each polymeric unit allow the formation of a stable three-dimensional network of cellulose-based derivatives by physical or chemical cross-linking. Physical cross-linking methods for cellulose-based materials include freeze–thaw [69], self-assembling [70], instantaneous gelation [71], reconstitution [72], inverse emulsion technique [73], and ionotropic gelation [74]. Chemical cross-linking can be achieved through chemical reactions [75], polymerization [76], or radiation (gamma, microwave, ultraviolet) [77]. Some derivatives of cellulose that are used for the synthesis of cellulose-based hydrogels include esters, ethers, and composites (IPN or polymeric blendings). Gels derived from cellulose derivatives have been shown to be effective adsorbents for various contaminants [78,79,80,81], some of them being dyes (Table 3). Updated literature from the last decade indicated that CS and its derivatives (carboxymethylcellulose, hydroxypropylcellulose) grafted and cross-linked with various reagents (acrylic acid, 2-acrylamido-propanesulphonic acid, itaconic acid, polyacrylic acid) afforded the obtaining of new polymers having good dye adsorption properties. The addition of various fillers (MMT, graphitic carbon nitride, MoS_2_, sepiolite, BNTN, carboxylated graphene oxide) or magnetic nanoparticles (Fe_3_O_4_, graphene quantum dots) in the chemical structure of cellulose-based hydrogels increased their dye sorption capability [82,83,84,85,86,87,88,89,90,91,92,93,94,95,96,97,98,99,100,101].

Quaternized cellulose, modified with PAA by free radical polymerization, was utilized as an adsorbent for the removal of MB dye [82]. The adsorption of dye occurred through electrostatic interaction between the ammonium groups of the cellulose-based gel and the carboxylic groups of PAA. 

Cellulose nanofibrillated (CNF) grafted with poly([2-(acryloyloxy) ethyl] trimethylammonium chloride) (PClAETA)) was obtained by free radical polymerization [83]. MBA acted as cross-linking agent, while ammonium persulfate (APS) initiated the free radical reaction. Increasing the CNF concentration led to greater hydrogel swelling due to the increased number of carboxyl groups in the hydrogel. Adsorption studies with MO dye showed that dye removal efficiency reached approximately 96%. Additionally, optimizing pH value to neutral (pH = 7.64) enhanced the absorption capacity of MO dye. 

**Figure 7:**
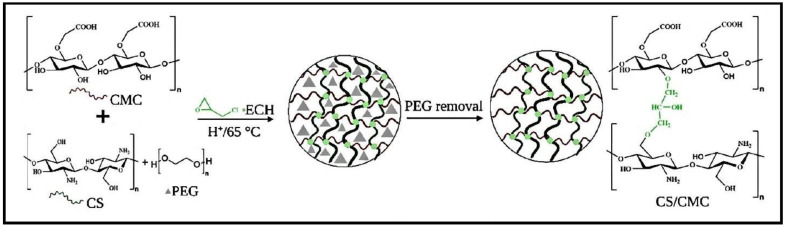
Preparation of CS/CMC-PEG hydrogels. Reproduced with permission from [84].

Hydrogels based on CS and carboxymethyl cellulose (CMC) were synthesized by chemically cross-linking with ECH and by using polyethylene glycol (PEG) as a pore-forming agent (Figure 7) [84]. The effectiveness of the CS/CMC hydrogels in removing CR and MB from water was assessed by considering factors such as pH, mass of the adsorbent, concentration of PEG additives, and initial dye concentrations. The adsorption process for CR followed the pseudo-second-order kinetics model, while MB followed the pseudo-first-order kinetics model. The adsorption isotherm for CR adsorption fitted to both Freundlich and Langmuir models, whilst the Langmuir model fitted better for the MB adsorption isotherm. The thermodynamic analysis demonstrated that adsorption of both dyes was spontaneous, with CR adsorption being endothermic and MB sorption being exothermic.

A new environmentally friendly dye adsorbent, tested for the removal of MB, was developed by combining hydroxypropyl cellulose (HPC) with molybdenum disulfide (MoS_2_) [85]. To achieve a more uniform dispersion of MoS_2_ nanosheets in HPC, esterification was used to attach MoS_2_ to polysaccharide chains. HPC-MoS_2_/HPC hydrogels exhibited superior adsorption performance for MB compared to HPC alone. Furthermore, the unique photo-catalytic properties of MoS_2_ allowed the recyclability and reusability of the sorbent through illumination, HPC-MoS_2_/HPC gel being easily activated with minimal loss of dye adsorbing capacity. 

New magnetic superabsorbent hydrogel nanocomposites were synthesized by incorporating magnetic iron oxide nanoparticles into CMC grafted with poly(acrylic acid) (PAA) in a single-step reaction [86]. The resulting nanocomposites were capable of removing Crystal violet (CV) from aqueous solutions. The adsorption data fitted well with the Redlich–Peterson isotherm model.

Quantum dots (QPs), having CdS in their structure, were integrated into a CMC-g-P(AA-g-AMPS) hydrogel matrix and employed for the removal of Rhodamine B (RhB) from aqueous solutions [87]. QPs are semiconductor nanoparticles with unique optical and electronic properties. The inclusion of CdS-QPs improved the thermal stability of the polymeric network. Desorption studies showed effective regeneration capability at various temperatures. After five cycles of adsorption–desorption, dye removal ranged from 95 to 75%. 

Graphitic carbon nitride (g-C_3_N_4_) reinforced an eco-friendly adsorbent made of sugarcane cellulose (SBC), and sodium carboxymethylcellulose (NaCMC), g-C_3_N_4_@SBC/CMC, was successfully prepared using a simple sol-gel technique [88]. The adsorption process of MB was accurately described by the pseudo-second-order kinetic model and Langmuir sorption model. Furthermore, the stability and reusability of the adsorbent were excellent, with almost no decline in adsorption capacity observed even after seven cycles. 

Sodium CMC-based hydrogel grafted with the copolymer of acrylic acid (AA) and itaconic acid (IA), coded as CMC-g-poly (AA-co-IA), was successfully synthesized for the removal of safranin-O from wastewater [89]. The swelling and removal efficiencies of CMC-g-poly (AA-co-IA) were improved by incorporating MMT clay nanosheets (CMC-g-poly (AA-co-IA)/MMT). The adsorption study indicated that both CMC-g-poly (AA-co-IA) and CMC-g-poly (AA-co-IA)/MMT followed a pseudo-second-order model for kinetic behavior, and the equilibrium data were well-fitted to the Langmuir isotherm model. Thermodynamic parameters indicated that the safranin-O adsorption by CMC-g-poly (AA-co-IA)/MMT is a spontaneous, exothermic, and entropy-decreasing process.

**Figure 8:**
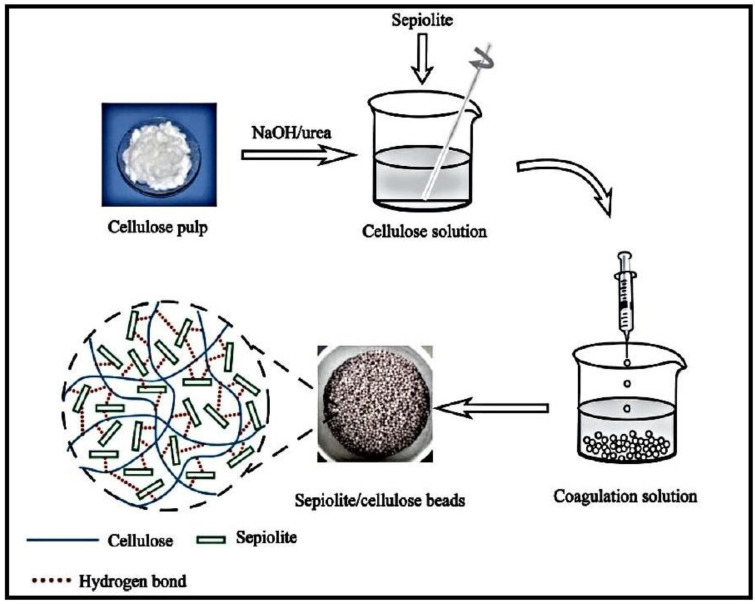
Schematic illustration for the preparation of Sep/cellulose hybrid beads. Reproduced with permission from [99].

An organic-inorganic hybrid adsorbent based on cellulose and sepiolite (Sep), a fibrous magnesium hydrosilicate, was synthesized and used for the elimination of Malachite green (MG) dye (Figure 8) [99]. The hybrid was formed by adding pretreated Sep to cellulose dispersed in a NaOH/urea aqueous solution, followed by the dropping of the mixture into a diluted HCl-CaCl_2_ solution. The resulting hydrogel beads demonstrated great thermal strength because of the thermal isolation effect of Sep molecules. 

Hydrogels, made of CMC-AM-GO and prepared using free radical polymerization by using different CMC percents, were employed for the removal of Acid Blue 133 (AB 133) from aqueous solutions [100]. The swelling kinetic data suggested that the gels exhibited a super Case II diffusion transport mechanism. The elimination capability varied depending on the percentage of GO in the hydrogels. 

**Figure 9:**
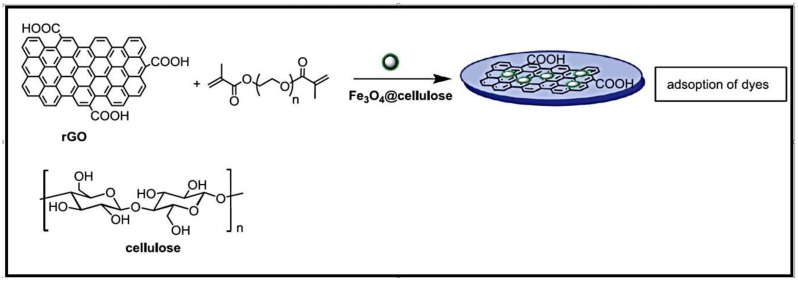
Synthesis of magnetic rGO-loaded PEGDMA-based hydrogels. Reproduced with permission from [101].

Magnetic hydrogels were synthesized by incorporating rGO and cellulose modified with magnetic nanoparticles into poly(ethylene glycol) dimethacrylate (PEG DMA)-based hydrogels by photo-polymerization (Figure 9) [101]. Cellulose, containing magnetic nanoparticles, was prepared by coprecipitation reaction of Fe^2+^ and Fe^3+^ in alkaline medium, followed by postcoating with the polysaccharide and rGO. The formed composites demonstrated high effectiveness in removing MB dye. The magnetic rGO-loaded hydrogel exhibited a high thermal stability, and could be regenerated without any reduction in its adsorption ability. 

Remarkable results concerning dye sorption by cellulose-based hydrogels (Table 3) can be observed for polymers having hydrophilic (co)polymers containing AA grafted onto soluble derivatives of the polysaccharide such as CMC [99], HPC [86], or acryloyl-cellulose [90] due to rapid swelling and exceptional adsorption capacity, which justifies their appellation as superabsorbents. Incorporation in these polymeric structures of MMT [99] or MoS_2_ [86] improved their dye absorptivity due to the occurrence of specific inorganic stack atomic layers of the filler.

#### 2.1.3. Starch-Based Hydrogels

Starch (ST) is a plentiful and inexpensive biopolymer that is biocompatible and biodegradable. It is found in plants such as wheat, maize, and rice, serving as a reserve carbohydrate. Granules of ST are composed of a combination of semicrystalline and soluble amylose (20–30%) and highly crystalline and insoluble amylopectin (70–80%). Amylose (Table 1) is made up of linear α-D-glucose units connected by α(1→4) glycosidic bonds, while amylopectin (Table 1) is composed of heavily branched α-D-glucose units linked by α(1→4) or α(1→6) glycosidic bonds [102]. 

Chemical modification of the polysaccharide can resolve the lack of adsorption ability of native ST. The literature from the last ten years indicated that grafting and cross-linking of ST and its derivatives (carboxymethylstarch, hydroxypropyl sulfate starch) with various reagents (acrylamide, 2-acrylamido-propanesulphonic acid, polyvinylimidazole) afforded the obtaining of hydrogels based on ST having dye adsorption properties. Furthermore, the integration of different fillers (GO, hydroxyapatite) or magnetic nanoparticles (Fe_3_O_4_) in chemical structure of hydrogels based on ST allowed the improving of the gel’s adsorption capacity (Table 4) [103,104,105].

Instantaneous gelation in boric acid of carboxymethyl starch-g-polyvinyl imidazole (CMST-PVIm), along with a combination containing PVA and Fe_3_O_4_, followed by cross-linking with glutaraldehyde (GA), resulted in the formation of magnetic nanocomposite hydrogel beads [106]. These hydrogel beads were utilized in adsorption studies for CV and CR dyes. Thermodynamic investigations indicated that the chemisorption process was spontaneous and endothermic. 

**Figure 10:**
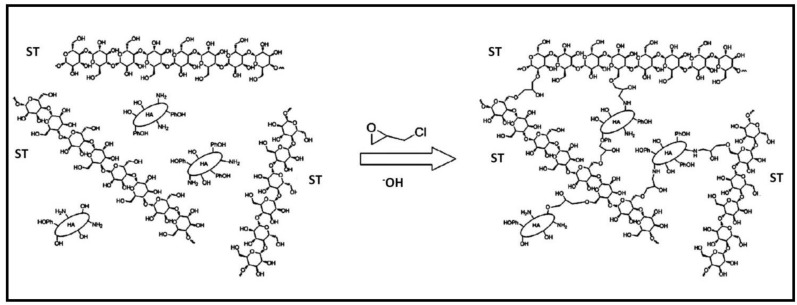
The synthesis mechanism of ST-HA. Reproduced with permission from [107].

Through the inverse suspension cross-linking method utilizing ECH as cross-linker, hydrogel beads composed of ST and humic acid (HA) were successfully fabricated (Figure 10) [107]. These composite beads were then utilized in studies focusing on the adsorption of MB dye, which was effectively retained on the composite gel through a combination of π–π interactions and ion exchange mechanisms. The composite demonstrated a robust regeneration capacity, reaching 92% after undergoing five cycles of adsorption–desorption. 

Hydroxypropyl sulfate starch (HPSST), obtained from 2-hydroxy-3-chloropropyl sulfate and ST in NaOH with ECH employed as cross-linking agent, exhibited effectiveness as adsorbent for the removal of MB dye. Its efficacy was attributed to favorable sorption outcomes and its notable capacity for full regeneration [108].

A porous nanocomposite hydrogel, based on ST, was created through the free radical copolymerization of AM and 2-acrylamido-2-methylpropane sulfonic acid (AMPS) onto the polysaccharide. This process was conducted in the presence of CaCO_3_ and GO [109]. The resulting gel found application in the removal of MB from aqueous solutions. CaCO_3_ particles served as porogen agents, and after synthesis, these inorganic solid nanoparticles were eliminated by solving the gel in an HCl solution. The adsorption ability for MB was enhanced due to the increased hydrogel porosity. Even after undergoing five cycles of adsorption–desorption, the removal efficiency for MB remained high at 95.4%. 

**Table 4:** Dye elimination results of hydrogels based on ST.

Adsorbent	Dye	Dye Retaining/Removal Capability	Reference
(CMST-g-PVIm/PVA/Fe_3_O_4_)	CV	91.58 mg/g	[106]
	CR	83.66 mg/g	
Fe_3_O_4_/ST-*g*-PAA	MV	31.847 mg/g	[110]
ST phosphate	CV	99% dye removal	[111]
Succinylated ST	MO	20% dye removal	[112]
	MB	83% dye removal	
ST@PAA	MB	133.65 mg/g	[113]
	CR	64.73 mg/g	
ST/HA	MB	111.10 mg/g	[107]
ST-g-P(AA-co-AM)/PDA	MB	2276 mg/g (pH 9)	[114]
ST-based sulfonic ion exchange resin	MB+RhB+MG	84.04% decolorization	[115]
ST-g-P(AMPS-co-DMAEMA)/benzyl chloride	BV 7	600 mg/g	[116]
MST	MB	70 mg/g	[117]
GST		75 mg/g	
VST		81 mg/g	
succinylated ST	MB	84 mg/g	[118]
ST-g-P(AA-co-AMPS)/GO	MB	769.23 mg/g	[109]
ST-g-PAM/GO/HAp	MG	297 mg/g	[119]

The nanocomposite consisting of ST-g-PAM/GO/HAp was used for the removal of MG dye [119]. The nanocomposite was synthesized by free radical copolymerization in the presence of GO nanosheets and varying amounts of HAp. Thermogravimetric analysis indicated that HAp nanoparticles acted as thermal barriers, resulting in higher initial decomposition temperatures when incorporated into the nanocomposite structure. The efficiency of desorption increased as the amount of HAp decreased within the gel structure. The addition of HAp to the sorbent matrix reduced the free spaces and porosity of the network. Consequently, after five cycles of adsorption–desorption, 25%, 17%, and 13% of MG were delivered from hydrogel nanocomposites with porosity levels of 11%, 25%, and 30%, respectively. 

Table 4 shows that the greatest results regarding dye absorption by starch-based hydrogels can be seen for superabsorbent polymers that included hydrophilic (co)polymers containing AA grafted onto the polysaccharide, followed by either coating [114] or incorporating [119] into the polymeric structure of universal surface modifiers such as PDA and GO nanolayers, respectively.

#### 2.1.4. Pullulan-Based Hydrogels

Pullulan (PUL), a biocompatible and nontoxic polysaccharide, is composed of maltotriose units joined by α(1–4) glycosidic bonds, while consecutive maltotriose moieties are connected by α(1–6) glycosidic bonds (Table 1). This polysaccharide and its derivatives have various uses in biomedicine, pharmaceuticals, food industry, and electronics. Literature that has been published over the past ten years showed that certain PUL derivatives have been utilized as adsorbents for various dyes in the treatment of wastewater, after the grafting and cross-linking of native polysaccharide with different reagents (polyacrylamide, poly(acrylic acid). The addition of various fillers (GO, activated carbon, MMT, polydopamine) in the obtained gel’s structure increased their dye sorption properties (Table 5). 

**Figure 11:**
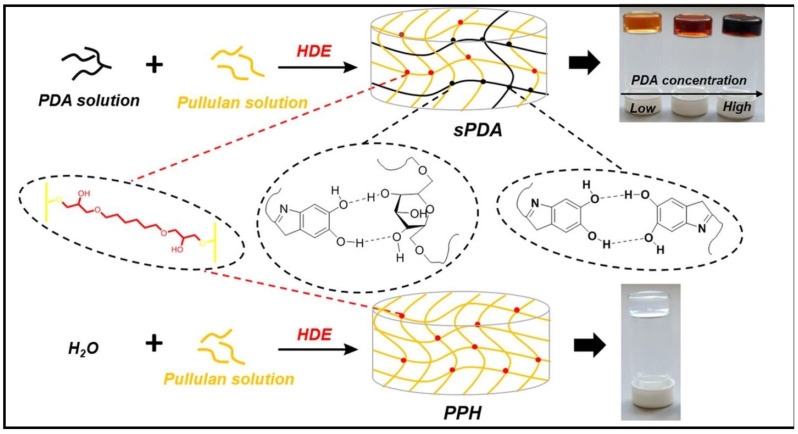
The syntheses of PPH and sPDA. Reproduced with permission from [120].

A semi-IPN polysaccharide hydrogel (PUL/PDA/HDE, also called sPDA), comprising PUL, polydopamine (PDA), and 1–6-hexanediol diglycidyl ether (HDE) as cross-linker, were used for the elimination of CV dye (Figure 11) [120]. PUL/PDA/HDE hydrogels exhibited improved thermostability, adjustable pore diameter, and enhanced swelling ability compared to pure PUL hydrogel (PPH) obtained by the cross-linking of the polysaccharide with HDE. Langmuir and pseudo-second-order models fitted well to sorption process of sPDA. After four cycles of adsorption experiments, the adsorption capacities still maintained 94%.

The composite hydrogel (PUL/PAM/GO), composed of PAM as carrier, grafted on PUL and modified with GO, was used for the elimination of MB dye [121]. The removal rate of MB by PUL/PAM/GO was 83.2% of dye in 140 min. Pseudo-second-order reaction, and the Langmuir model best described the adsorption process. Thermodynamic studies revealed that the adsorption was exothermic and spontaneous. 

PUL/PAA/Activated Carbon (PUL/PAM/AC) hydrogel was utilized as sorbent for MB dye adsorption [122]. The hydrogel comprised potassium persulfate (KPS) as initiator, MBA as cross-linking agent, and sodium hydroxide as activator. The optimal ratio of PUL to AC was obtained as 6:1. The adsorption of MB obeyed pseudo-first-order kinetic and Langmuir isotherm models. 

A composite hydrogel (PUL/PDA/MMT), obtained by incorporation of PDA and MMT into PUL structure, was employed for CV adsorption [123]. By adjusting the mass ratio of PDA/MMT, the adsorbent’s properties can be improved. The adsorption data were best described by a Langmuir isotherm and pseudo-second-order kinetic model.

The chemical cross-linking of PUL with diglycerols, such as ECH, 1,2-bis(2,3-epoxypropoxy)-ethane (BEPE) and tetramethylene glycol diglycidyl ether (TGDE), was the first step in the obtaining of PUL-based nanocomposite employed for the removal of CV dye [124]. TGDE (longest chain length)-derived gel, having the best performance (acceptable porous structure, good swelling ability, and strong rigidity), was the polysaccharide matrix chosen for the incorporation of MMT, which afforded the acquirement of nanocomposites with improved characteristics. Adsorption behavior was well represented by pseudo-second-order kinetic and Langmuir isotherm models. 

Pullulan-graft-polyacrylamide (PUL-g-PAM) hydrogel, prepared by radical polymerization with MBA as cross-linking agent, was utilized as sorbent for removal of MB and Reactive blue 2 (RB 2) dyes [125]. Langmuir and Freundlich isotherms and pseudo-second-order models fitted well with the experimental data, whilst thermodynamic studies showed that adsorption of both dyes was an endothermic and spontaneous process. 

#### 2.1.5. Dextran-Based Hydrogels

Dextran (Dex) is a biocompatible and nontoxic polysaccharide. Its main chain is composed of α-D-glucopyranose units which are linked by linear α-1,6 glycosidic bonds, with a reduced degree of α-1,3-linked side chains (Table 1). Literature in the past decade indicated that dye adsorbing systems based on Dex hydrogels were obtained after the amination of cross-linked polysaccharide with a mixture of epichlorohydrin and a tertiary amine, or by treating glycidyl methacrylate substituted Dex with acrylic acid (Table 5). Other applications of Dex-based hydrogels include biomedical, pharmaceutical, food, and chemical industries.

**Figure 12:**
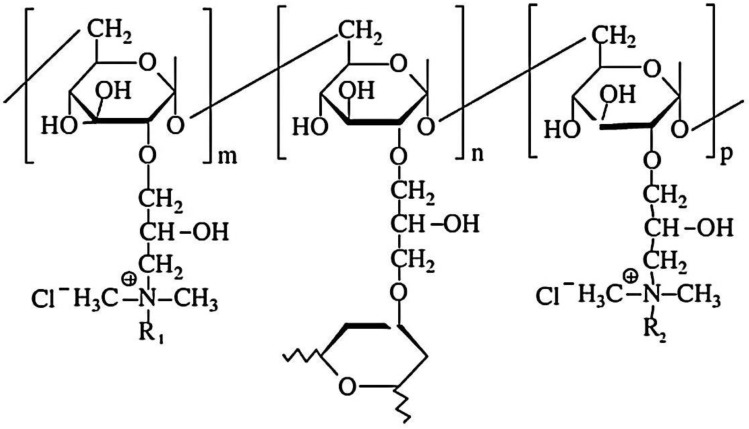
The chemical structure of amphiphilic dextran gels (R_1_ = C_12_ (dodecyl) or C_16_ (hexadecyl); R_2_ = C_2_ (ethyl)) (adapted and redrawn from [126]).

**Figure 13:**
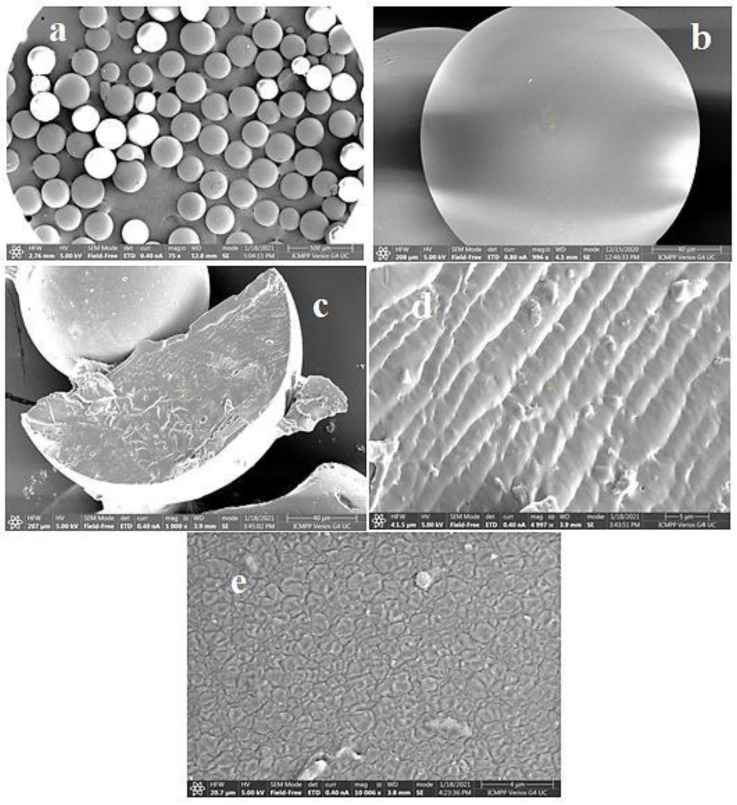
SEM images of dextran microspheres, magnified 100 times (**a**) and 1000 times (**b**); dextran microparticles in cross-section, increased 1000 times (**c**) and 5000 times (**d**); dextran gel surface, magnified 10,000 times (**e**). Reproduced with permission from [127].

Cationic hydrophobically modified Dex-based hydrogels (Dex-Q1) (Figure 12), having as side-chains quaternary ammonium chloride groups with different polarities and synthesized with different molar ratios between hydrophilic (R_2_ = C_2_) and hydrophobic groups (R_1_ = C_12_/C_16_), total content in amino groups (50–68 mol%) and water retention capacity (3–15 g water/g dry hydrogel), were tested as sorbents of MO and Rose Bengal (RB) dyes [126]. Examined in their dry state, the polymeric microparticles exhibited a perfectly spherical shape with the diameters ranging from 100 to 220 μm [127,128]. When observed at a magnification of up to 1000 (Figure 13a,b), their surface appeared flattened. At a magnification of 10,000 (Figure 13e), the surface exhibited creases, a feature resulting from the gradual dehydration which determined the contraction of the surface layer during the drying process. Both the surface and cross-section of the microspheres were free of pores (Figure 13a–e). These findings confirm the absence of porosity in the dry state of Dex-based hydrogels, a characteristic previously established for other dextran-based microparticles obtained without using porogen agents. The rate of adsorption was mostly influenced by the porosity of the gel and dye’s molecular weight, and the affinity between the polymer and dye was improved by higher levels of hydrophobic groups. MO adsorption was a little affected by increased lipophilicity, while the amount of adsorbed RB decreased considerably with enhanced hydrophobicity. By analyzing adsorption data obtained from kinetic and thermodynamic analysis, it was determined that dye sorption was spontaneous and thermodynamically advantageous, the process being influenced by both diffusion and ion exchange. RB retention ability by Dex-Q1 was very high, comparable with that of a superabsorbent polymer, due to polymeric hydrophilicity and high swelling porosity (Table 5). 

An amphiphilic cationic Dex-based hydrogel (Dex-Q2), with two types of quaternary ammonium pendant groups with different polarities and having the same molar ratio between hydrophilic and hydrophobic side-chains, was synthesized and examined as adsorbent for various dyes (MO, Indigo Carmin (IC), Orange II, RB) [129]. The Langmuir model best described the adsorption equilibrium, whilst the Freundlich model was suitable when 40–70% of the cationic sites of the gel were occupied by dye molecules. A pseudo-second-order kinetic model fitted well with sorption data, and thermodynamic studies indicated that adsorption process consisted of diffusion for RB, chemisorption for IC, and a combination of physical and chemical interactions for MO and Orange II. The adsorption ability of Dex-based hydrogel was greater, for the same dyes, in comparison with inorganic–polymer hybrids. A quick and complete regeneration of the cross-linked polymer was acquired by employing a successive addition of water, NaCl 0.5 M, and methanol. 

A biocompatible superabsorbent hydrogel, Dex-GMA/PAA, was prepared via copolymerization of glycidyl methacrylate substituted dextran (Dex-GMA) with hydrophilic AA, and was tested as sorbent for the removal of MB and CV dyes with the best results between dextran-based hydrogels (Table 4) [130]. Dex-MA/PAA hydrogel showed a fast adsorption rate and the elimination efficacy of MB and CV reached 93.9% and 86.4%, respectively, within 1 min at an initial concentration of 50 mg/L. The adsorption equilibrium data fitted the Sips isotherm model, with dye adsorption occurring efficiently over a large pH range (3–10) and in a wide temperature interval (20–60 °C). The removal efficiencies for both dyes were higher than 95%, even after five adsorption–desorption cycles.

**Table 5:** Dye removal ability of pullulan and dextran-based hydrogels.

Adsorbent	Dye	Dye Retention/Removal Capacity	Reference
PUL/PDA/HDE	CV	108 mg/g	[120]
PUL/PAM/GO	MB	438.7 mg/g	[121]
PUL/PAM/AC	MB	591.4 mg/g	[122]
PUL/PDA/MMT	CV	112.45 mg/g	[123]
PUL/MMT	CV	80 mg/g	[124]
PUL-g-PAM	MB	386.81 mg/g	[125]
	RB 2	273.24 mg/g	
PUL-g-PAPTAC	*Azocarmine B*	113.63 mg/g	[131]
Dex-Q2	MO	705 mg/g	[129]
	IC	732 mg/g	
	Orange II	652 mg/g	
	RB	654 mg/g	
Dex-Q1	MO	893 mg/g	[126]
	RB	1718 mg/g	
Dex-GMA/PAA	MB	1994 mg/g	[130]
	CV	2390 mg/g	

### 2.2. The Effect of Various Factors on Adsorption Capacity

Dye adsorption depends on different parameters such as initial dye concentration, sorbent amount, contact time, dye type, and pH value. In what follows, the influence of each factor on dye adsorption is analyzed. 

#### 2.2.1. Initial Dye Concentration

**Figure 14:**
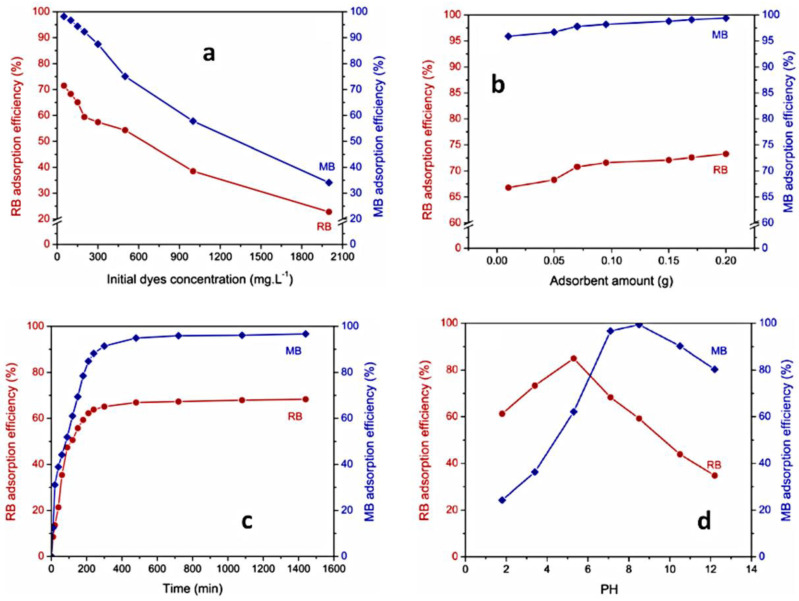
Variation of dye sorption versus time, as a function of initial dye concentration (**a**), absorbent amount (**b**), contact time (**c**), and pH (**d**). Reproduced with permission from [125].

The increase in adsorption quantity of dyes with the augmentation of their initial concentration can be attributed to a high presence of dye molecules near the surface adsorbent and a stronger force driving mass transfer, prior to reaching the equilibrium between adsorption and desorption. At the same time, the percent of dye removal decreased, which may be caused by the saturation of adsorption sites of the adsorbent. The described behavior can be observed for PUL-g-PAM gel in the adsorption process of MB and RB dyes (Figure 14a) [125].

#### 2.2.2. Adsorbent Amount

The adsorption efficiency increases with the augmentation of sorbent amount due to the increasing of the sorption sites on the sorbent surface. The explained behavior can be observed for MB and RB dyes adsorption by PUL-g-PAM hydrogel (Figure 14b) [125].

#### 2.2.3. Contact Time

The removal of dye from aqueous solution displayed a rapid initial increase in adsorption, succeeded by a slowdown of the process until it reached an equilibrium state. In the beginning, dye molecules quickly adhered to the surface of the gel via mass transfer. However, the sorption process was subsequently delayed due to a decrease in available external sites of the hydrogel and the reduced diffusion of dye molecules to the internal sites of the polymeric matrix. When the number of sorption sites significantly decreased, the equilibrium state was achieved. The explicated behavior can be seen for PUL-g-PAM gel, used for removal of MB and RB dyes (Figure 14c) [125].

#### 2.2.4. Dye Type

The nature of dye affected adsorption efficiency. Dyes with high molar masses showed a limited adsorption by the hydrogel. Between dyes tested (MB, RB), RB had the lowest adsorption amount on PUL-g-PAM (Figure 14a–c) [125]. This was attributed to bulky structure and high molar mass of RB compared with MB [125]. Furthermore, when a sorbent has multiple functional groups within its chemical structure, it determined the binding of a greater number of its active sites with dye molecules, which diminished the amount of sorbed dye. RB dye serves as an example in this sense, as it contains two sulphonate groups within its chemical composition [129].

#### 2.2.5. pH

pH level plays a significant role in dye absorption, especially when the principal forces of sorbent–adsorbate are electrostatic in nature. Studies concerning MB and RB adsorption by PUL-g-PAM hydrogel [125] showed that at pH < 6, the hydrogel adsorbed RB more than MB, and at pH > 6, the amount of MB was higher than that of RB, the explanation for the modification of trend sorption efficiency for both dyes being pHZPC value of 6.12 determined for PUL-g-PAM (Figure 14d). At a pH < 6.12, the adsorbent surface becomes positively charged, which affords the electrostatic attraction between polymeric sites and sulphonate groups of the RB dye while in the same pH range; MB dye is neutral. Hence, the higher adsorption of RB compared to MB, for this pH range, is rational. At pH > 6.12, the negatively charged sites on PUL-g-PAM surface afforded a strong electrostatic attraction with cationic MB, which leads to an increase in the adsorption capacity of MB compared to RB. 

### 2.3. Adsorption Kinetics

Adsorption kinetics describe the rate at which solute is adsorbed and the resident time of the adsorbates on the solid–liquid interface. There are four stages in the adsorption process [132,133]. In the first stage, dye molecules move from the bulk liquid phase to the boundary layer. The second stage involves the diffusion of the adsorbate from the boundary layer to the external surface of the adsorbent (external diffusion). The third stage consists of transportation of dye molecules into the pores of the adsorbent (internal diffusion). The final stage involves interactions between dye molecules and the active sites of sorbent. Several kinetic models, including pseudo-first-order, pseudo-second-order, and intraparticle diffusion, are used to study adsorption process. 

#### 2.3.1. Pseudo-First-Order Model (PFOM)

The Lagergren model, also known as the PFOM, considers that the predominant mechanism in the adsorption process is the mass transfer between solution and solid phase [134]. The degree of adsorption is directly related with the difference between the amount of dye adsorbed on the gel at equilibrium time and a specific time. Equations (1) and (2) represent linear and nonlinear forms of the PFOM.

(1) $$\log\left(q_{exp}-q_{t}\right)=logq_{exp}-\frac{k_{1}t}{2.303}$$

(2) $$q_{t\;}=q_{exp\;}\left(1-e^{-k_{1}t}\right)$$

where *t* is the contact time (min); *k*_1_ (min^−1^) is PFOM rate constant; *q_exp_* (mmol/g) is dye adsorbed amount at equilibrium; and *q_t_* (mmol/g) is dye sorbed quantity at moment *t.*

#### 2.3.2. Pseudo-Second-Order Model (PSOM)

PSOM, also known as the Ho and McKayrate equation model, is frequently associated with the chemisorption process in which there is a sharing or exchange of electrons between adsorbent and sorbate [134]. Linear and nonlinear forms of PSOM are displayed in Equations (3) and (4).

(3) $$\frac{t}{q_{t}}=\frac{1}{k_{2}q_{exp}^{2}}+\frac{t}{q_{exp}}$$

(4) $$q_{t}=\frac{k_{2}q_{exp}^{2}t}{1+k_{2\;}q_{exp}t}$$

where *t* (min), *q_exp_* (mmol/g), and *q_t_* (mmol/g) have the same meaning as described before; *k*_2_ (g/mmol min) is PSOM rate constant.

#### 2.3.3. Intraparticle Diffusion Model (IDM)

The equation of IDM, developed by Weber and Morris, is shown in Equation (5) [129]. This model is frequently utilized to assess the sorption diffusion mechanism.

(5) $$q_{t}=k_{id}\sqrt{t}+C$$

where *k_id_* (mmol/g min^0.5^) is the rate constant for the IDM, while *C* (mmol/g) is a parameter directly proportional with the boundary layer thickness; *t* (min) and *q_t_* (mmol/g) have the same significance as discussed previously.

### 2.4. Adsorption Isotherms

The adsorption isotherms offer the relationships between sorbate concentration held on the solid phase and its concentration in solution at equilibrium. These isotherms also show potential interactions between sorbate and adsorbent. Two-parameter model isotherms such as Langmuir, Freundlich, Dubinin–Raduskevich, and three-parameter models such as Sips and Hill are often used to evaluate and compare the adsorption efficiency of different adsorbents. 

#### 2.4.1. Langmuir Model (LM)

The LM assumes that there is a homogeneous monolayer sorption on adsorbent binding sites that are all equivalent to each other [127]. The linear and nonlinear forms of the LM are described in Equations (6) and (7).

(6) $$\frac{C_{eq}}{q_{exp}}=\frac{C_{eq}}{Q_{L}}+\frac{1}{K_{L}Q_{L}}$$

(7) $$q_{exp}=\frac{Q_{L}K_{L}C_{eq}}{1+K_{L}C_{eq}}$$

where *q_exp_* (mmol/g) has the same meaning as shown above; *C_eq_* (mM) is adsorbate concentration at equilibrium; *Q_L_* is maximum sorbent capacity (mmol/g); and *K_L_* (L/mmol) is Langmuir equilibrium constant. *R_L_*, a nondimensional separation factor, is another parameter of the Langmuir equation (Equation (8)).

(8) $$R_{L}=\frac{1}{1+K_{L}C_{i}}$$

where *C_i_* (mM) is the initial concentration of sorbate. *R_L_* values states the adsorption efficiency. So, sorption can be unfavorable (*R_L_* > 1), linear (*R_L_* = 1), favorable (0 < *R_L_* < 1), or irreversible (*R_L_* = 0).

#### 2.4.2. Freundlich Model (FM)

FM takes into account a reversible, nonideal, and multilayer adsorption that occurs on heterogeneous surfaces. FM linear and nonlinear forms are illustrated in Equations (9) and (10) [127].

(9) $$lnq_{exp}=lnK_{F}+\frac{1}{n_{F}}lnC_{eq}$$

(10) $$q_{exp\;}=K_{F}C_{eq}^{1/n_{F}}$$

where *q_exp_* (mmol/g) and *C_eq_* (mM) have the same meaning as discussed before; *K_F_* (mmol/g) is Freundlich equilibrium constant. *n_F_*, the heterogeneity factor, indicates adsorption type and sorbate sites heterogeneity. If 0 < 1/*n_F_* < 1, the adsorption is favorable.

#### 2.4.3. Dubinin–Radushkevich Model (D-RM)

D-RM considers that the adsorption energy has a Gaussian distribution on the heterogeneous surfaces [127]. Linear and nonlinear forms of D-RM are revealed in Equations (11) and (12).

(11) $$lnq_{exp}=lnQ_{RD}-\beta \varepsilon^{2}$$

(12) qexp=QRDexp{−β[RTln(1+1Cexp)]2}

where *q_exp_* (mmol/g) has the same meaning as described previously; *Q_DR_* is maximum adsorbent capacity (mmol/g). *β* (mol^2^/J^2^) is a constant connected with the mean free energy per molecule of adsorbate for shifting from its place in the solution to infinity, and *ε* is Polanyi potential (Equation (13)).

(13) $$\varepsilon =RTln\left(1+\frac{1}{C_{eq}}\right)$$

where *R* is the universal gas constant (8.314 J/mol K) and *T* is the solution temperature expressed in Kelvin scale (K).

D-RM allows one to determine the mean free energy of sorption for ligand molecules, *E* (kJ/mol) (Equation (14)). This factor is indispensable for knowing the chief forces of adsorption (physical or chemical ones). When *E* < 8 kJ/mol, physical forces control the sorption; when 8 < *E* < 16 kJ/mol, the adsorption mechanism relies on ion exchange, and if *E* > 16 kJ/mol, the adsorption is handled by chemisorption.

(14) $$E=\frac{1}{\sqrt{2\beta }}$$

#### 2.4.4. Sips Model (SM)

SM, employed for adsorption onto heterogeneous surfaces, is a combined form of LM and FM [127]. This three-parameter model avoids the limitation of adsorbate concentration associated with FM. When *C_eq_* gets close to a low value, SM reduces to FM, while at high *C_eq_*, it approaches LM. Linear and nonlinear forms of SM are shown in Equations (15) and (16).

(15) $$ln\left(\frac{q_{exp}}{Q_{S}-q_{exp}}\right)=n_{S}\left(lnC_{eq}+lnK_{S}\right)$$

(16) $$q_{exp}=\frac{Q_{S}\;{\left(K_{S}C_{eq}\right)}^{n_{S}}}{1+{\left(K_{S}C_{eq}\right)}^{n_{S}}}$$

where *q_exp_* (mmol/g) and *C_eq_* (mM) have the same meaning as shown before; *K_S_* (L/mmol) is Sips equilibrium constant; *n_S_* is the heterogeneity Sips factor, while *Q_S_* is the maximum adsorbent capacity (mmol/g).

#### 2.4.5. Hill Model (HM)

HM considers that adsorption onto homogeneous surfaces is a cooperative process where ligands can bind at one site of absorbent and thus, this initial binding can potentially affect other binding sites on the same macromolecule [127]. Linear and nonlinear forms of HM are revealed in Equations (17) and (18).

(17) $$log\frac{q_{exp}}{Q_{max,H}-q_{exp}}=n_{H}log\left(C_{eq}^{n_{H}}\right)-log\left(K_{H}\right)$$

(18) $$q_{exp}=\frac{Q_{max,\;H}C_{eq}^{n_{H}}}{K_{H}+C_{eq}^{n_{H}}}\;$$

where *q_exp_* (mmol/g) and *C_eq_* (mM) have the same meaning as discussed previously; *Q_max,H_* (mmol/g) is the maximum adsorbent capacity; K_H_ (mmol/L) is Hill equilibrium constant, while Hill coefficient, *n_H_*, is the measure of binding cooperativity. Binding can be positive cooperative (*n_H_* > 1), noncooperative (*n_H_* = 1), or negative cooperative (*n_H_* < 1).

### 2.5. Thermodynamic Parameters

Enthalpy change (ΔH°), Gibbs free energy change (ΔG°), and entropy change (ΔS°) are the thermodynamic parameters that offer crucial information about the spontaneity and endo/exothermic nature of sorption processes [129]. ΔG° value is determined by Equation (19).

(19) ΔG°=−RT lnKc

where K_c_ is the distribution constant, R is the universal gas constant (8.314 J/mol_K), and T is the absolute temperature (K). Negative ΔG° values indicate that dye adsorption is favorable and spontaneous. Van’t Hoff equation (Equation (20)) provides the relationship between ΔS° and ΔH°.

(20) ln Kc=ΔS°/R−ΔH°/RT

where Kc, R, and T have the same meaning as above.

Negative ΔH° values indicate an exothermic sorption, while positive ΔH° values reveal an endothermic adsorption. ΔS° is proportional to the degree of disorder at the interface of solid–liquid during dye adsorption. The higher the degree of disorder, the higher the entropy.

### 2.6. Reusing of Adsorbents Based on Natural Polysaccharide Hydrogels 

The regeneration of sorbents is crucial, as it aids in decreasing the overall cost of treating wastewater and the amount of waste produced. An added advantage of studying the recycling of gels is the improved understanding of the sorption mechanism. Exploring desorption is the foundation for researching the reusability of gels. Consequently, numerous cycles of adsorption–desorption are conducted, with a fresh dye solution used each time, and the sorbent being washed and dried after each sorption process. The sorbent’s recycling ability is determined by the number of cycles after which the hydrogel’s adsorption capacity remains close to its original value. This is typically measured by the regeneration efficiency (*RE*, %), which is the ratio of the sorption performance in the *n*-th cycle (*q_n_*, mg/g) to the sorption capacity in the first cycle (*q*_1_, mg/g) (Equation (21)).

(21) $$RE\;\left(\%\right)=\frac{q_{n}}{q_{1}}\times 100$$

*RE*% is related to the working life cycle of hydrogels and is important when aiming to translate the sorbent to pilot scale or industrial level use.

## 3. Conclusions and Future Prospectives

Water pollution is a significant problem worldwide, with the treatment of industrial and urban wastewater being a top priority. Therefore, finding an effective method to remove dyes from wastewater is a major challenge. Adsorption techniques has proven successful in removing dyes, being a simple, effective and cheap method. Natural polysaccharide-based hydrogels have gained attention in the last decades because their ability to eliminate pollutants from wastewater and the capacity to be eco-friendly due to their biodegradable and nontoxic nature. This review provides a summary of the progress made in the last decade, in the field of natural polysaccharide gels for the management of dye-contaminated wastewater. These hydrogels based on chitin, chitosan, cellulose, starch, pullulan, and dextran demonstrate the ability to adsorb and selectively remove dyes, the influence of their different surface functionalities also being presented in this review. The incorporation of additives (fillers, magnets) in hydrogel structures afforded the enhancement of their properties in dye removal. Thus, the addition of nanofillers such as GO, BNTN, HAp, or MMT, which create compact cross-linked structures, allowed the increase of hydrogel’s mechanical durability and sorption ability. Additionally, magnetic biobased gels offer the advantage of being able to separate and restore the sorbents using magnets. Certain hydrogels show remarkable dye adsorption properties without the addition of additives. These cross-linked polymers are called superabsorbent gels and the adding of nanofillers and/or magnets in their structure greatly improved the sorption results. This review also discussed the impact of various factors (initial dye concentration, sorbent amount, contact time, dye type, and pH value) on the ability of hydrogels to retain dyes. Additionally, it presented commonly used kinetic (PFOM, PSOM, IDM) and isotherm models (LM, FM, D-RM, SM, HM), as well as thermodynamic parameters (ΔH°, ΔG°, ΔS°) that are used in the study of dye adsorption processes.

Some problems, always encountered in dye elimination, are mentioned below, along with possible solutions.

➢ Following several treatment cycles, the adsorbents undergo a decline in their active sites and transform into waste, thereby causing additional pollution. Hence, it is crucial to address this problem in an environmentally-friendly manner. One potential solution is to utilize the used adsorbent for various purposes, such as catalysis, antimicrobial applications, or energy generation, in order to minimize or eliminate waste production.
➢ Most adsorption studies, performed with polysaccharide-based hydrogels as adsorbents, are conducted at certain pH values, initial adsorbate amounts, adsorption rates, and isotherms. To overcome these limitations, scientists have achieved spectral and computational studies aiming to clarify the detailed adsorption mechanism on atomic or molecular scale.

Numerous hydrogels based on natural polysaccharides have been efficiently synthesized and utilized to eliminate dyes. There are undoubtedly more to be developed in the coming future by taking into account the huge number of unexploited natural polysaccharides which, besides their biodegradability and nontoxic nature, are stable, abundant, and, most often, inexpensive.

## Figures and Tables

**Table 1:** Polysaccharides used in the synthesis of hydrogels utilized for dye removal.

Polysaccharide	Chemical Structure	Characteristics	Ref.
chitin	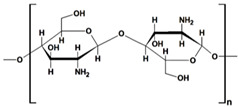	➢ unbranched homopolysaccharide; ➢ crystalline, with hydroxyl, amino, and acetyl groups; ➢ poor solubility in solvents	[42]
chitosan	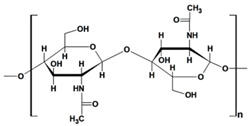	➢ unbranched homopolysaccharide; ➢ crystalline, cationic, with amino and hydroxyl groups; ➢ low solubility in many solvents, soluble in dilute acidic solutions; ➢ viscous polymer solution; ➢ excellent adsorption capacity and thermal stability	[43]
cellulose	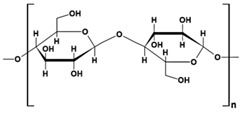	➢ unbranched homopolysaccharide; ➢ nonionic polysaccharide, semicrystalline, with hydroxyl groups; ➢ poor solubility in water, but soluble in organic solvents, alkali/urea aqueous media and ionic liquids	[44]
starch	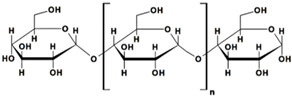 amylose 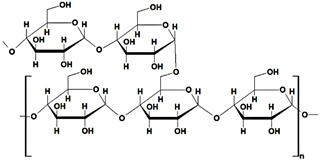 Amylopectin	➢ branched heteropolysaccharide; ➢ semicrystalline and nonionic polysaccharide, insoluble in cold water, alcohol or other solvents; ➢ consisted of linear amylose (20–30%, semicrystalline, soluble in hot water) and amylopectin (70–80%, crystalline, insoluble in hot water) with hydroxyl groups; ➢ swells in water at r.t. ➢ has a source-dependent structure	[45]
pullulan	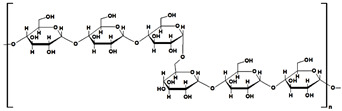	➢ linear and unbranched heteropolysaccharide; ➢ amorphous and nonionic with hydroxyl groups; ➢ great mechanical properties; ➢ water solubility with high chemical reactivity	[46]
dextran	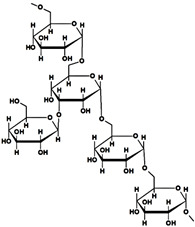	➢ branched homopolysaccharide; ➢ amorphous and nonionic, flexible structure with hydroxyl groups; ➢ soluble in water (95% linear linkage); ➢ form dextran sulfate and cationic dextran for produced various hydrogels	[47]

**Table 3:** Dye removal results of some recent cellulose-based hydrogels.

Adsorbent	Dye	Dye Retaining/Elimination Ability	Reference
cellulose-g-PAM	MB	734.816 mg/g	[82]
CMC-g-PAA	MO	82.0% elimination capacity	[90]
	DB BLN	77.4% elimination capacity	
	MG	95.3% elimination capacity	
acryloyl cellulose-g-PAA	MB	3003 mg/g	[91]
cellulose-g-P(AA-co-AM)	AB 93	82% elimination for both dyes	[92]
	MB		
cellulose-g-CDNMA	MB	15 mg/g	[93]
	MO	12 mg/g	
CNF-g-PClAETA	MO	1379.0 mg/g	[83]
CS/CMC-PEG	CR	1053.88 mg/g	[84]
	MB	331.72 mg/g	
HPC-based MoS_2_	MB	6153 mg/g	[85]
CMC/g-C_3_H_4_/ZnO	MV	96.43 mg/g	[94]
HPMC-g-PAM/NaMMT	CV	76% removal efficiency (five cycles)	[95]
lignocellulose-g-PAA/MMT	MB	1994.38 mg/g	[96]
CMC/PAA/Fe^3+^, Fe^2+^	CV	200 mg/L	[86]
CdS/QDs	RhB	137 mg/g	[87]
g-C_3_N_4_@SBC/CMC	MB	362.3 mg/g	[88]
CMC-g-P(AA-co-IA)	Safranin-O	185,185 mg/g	[89]
CMC-g-P(AA-co-IA)/MMT		191,205 mg/g	
cellulose/Sep	MG	314.47 mg/g	[99]
CMC/AM/GO	AB 133	185.45 mg/g	[100]
PVA/CMC/GO/BNTN	MB	172.14 mg/g (30 °C)	[97]
CMC/c-GO	MB	180.32 mg/g	[98]
rGO/cellulose/Fe^2+^, Fe^3+^/PEG DMA	MB	119 mg/g	[101]

## Data Availability

Not applicable.

## Abbreviations

**AA**	acrylamide	**IP6**	phytic acid (inositol hexaphosphate)
**AAM**	acrylamidomethylated	**KPS**	potassium persulfate
**AB 93**	Acid Blue 93	**MA**	methyl acrylate
**AB 113**	Acid blue 133	**MAM**	methacrylamide
**AMPS**	2-acrylamido-propanesulphonic acid	**MB**	Methylene Blue
**AO7**	Acid Orange 7	**MBA**	N, N-methylene bisacrylamide
**APS**	ammonium persulfate	**METAC**	2-(methacryloyloxyethyl)trimethylammonium chloride
**APTAC**	(3-acrylamidopropyl)trimethyl ammonium chloride	**MG**	Malachite green
**BEPE**	1,2-bis(2,3-epoxypropoxy)-ethane	**MMT**	montmorillonite
**BNTN**	bentonite	**MO**	Methyl Orange
**BV 7**	Basic Violet 7	**MST**	malonate starch
**c-GO**	carboxylated graphene oxide	**MV**	Methyl violet
**CDNMA**	acrylamidomethylated cyclodextrin	**NaCMC**	sodium carboxymethyl cellulose
**CMC**	carboxymethyl cellulose	**NaMA**	sodium methacrylate
**CMST**	carboxymethyl starch	**NaMMT**	sodium montmorillonite
**CN**	cellulose nanofibrillated	**NR**	Neutral Red
**CS**	chitosan	**NVIm**	N-vinylimidazole
**CR**	Congo Red	**PA**	polyacrylate
**CV**	Crystal violet	**PAA**	poly(acrylic acid)
**β-CD**	β-cyclodextrin	**PAM**	polyacrylamide
**DMAEMA**	dimethylaminoethyl methacrylate	**PAMPS**	poly-2-acrylamido-2-methylpropansulfonic acid
**DMAM**	dimethyl acrylamide	**PClAETA**	poly[2-(acryloyloxy)ethyl]trimethylammonium chloride
**DB 38**	Direct Black 38	**PDA**	polydopamine
**DB BlN**	Disperse Blue BLN	**PEG DMA**	poly(ethylene glycol) dimethacrylate
**DR 81**	Direct Red 81	**PNVIm**	poly N-vinylimidazole
**ECH**	epichlorohydrin	**PVA**	polyvinyl amine
**EDA**	ethylenediamine	**QDs**	quantum dots
**FY 3**	Food yellow 3	**RB**	Rose Bengal
**g-C_3_H_4_**	graphitic carbon nitride	**RB 2**	Reactive Blue 2
**GA**	glutaraldehyde	**RB 5**	Reactive Black 5
**GMA**	glycidyl methacrylate	**RB 221**	Reactive Blue 221
**GO**	graphene oxide	**rGO**	reduced graphene oxide
**GST**	glutarate starch	**RhB**	Rhodamine B
**HA**	humic acid	**RR 136**	Reactive red 136
**HAp**	hydroxyapatite	**SBC**	sugarcane cellulose
**HDE**	1–6-hexanediol diglycidyl ether	**Sep**	sepiolite
**HEST**	hydroxyethyl starch	**ST**	starch
**HPC**	hydroxypropyl cellulose	**TA**	tannic acid
**HPMC**	hydroxypropylmethyl cellulose	**TETA**	triethylenetetramine
**HPSST**	hydroxypropyl sulfate starch	**TGDE**	tetramethylene glycol diglycidyl ether
**IA**	itaconic acid	**VST**	valerate starch
**IC**	Indigo Carmin	**r.t.**	room temperature
